# The CTSA External Reviewer Exchange Consortium (CEREC): Engagement and efficacy

**DOI:** 10.1017/cts.2019.411

**Published:** 2019-10-02

**Authors:** Margaret Schneider, April Bagaporo, Jennifer A. Croker, Adam Davidson, Pam Dillon, Aileen Dinkjian, Madeline Gibson, Nia Indelicato, Amy J. Jenkins, Tanya Mathew, Renee McCoy, Hardeep Ranu, Kai Zheng

**Affiliations:** 1 Institute for Clinical and Translational Science, University of California, Irvine, CA, USA; 2 Center for Clinical and Translational Science, University of Alabama at Birmingham, Birmingham, AL, USA; 3 Institute of Translational Health Sciences, University of Washington, Seattle, WA, USA; 4 C. Kenneth and Dianne Wright Center for Clinical and Translational Research, Virginia Commonwealth University, Richmond, VA, USA; 5 Southern California Clinical and Translational Science Institute, University of Southern California, Los Angeles, CA, USA; 6 Translational Research Institute, University of Arkansas for Medical Sciences, Little Rock, AR, USA; 7 Center for Clinical and Translational Science, The Ohio State University, Columbus, OH, USA; 8 Clinical Translational Science Institute of Southeast Wisconsin, Medical College of Wisconsin, Milwaukee, WI, USA; 9 Harvard Catalyst, Harvard University, Boston, MA, USA; 10 Department of Informatics, School of Information and Computer Sciences, University of California, Irvine, CA, USA

**Keywords:** Peer review, CTSA, pilot study, collaboration, conflict of interest

## Abstract

**Introduction:**

Many institutions evaluate applications for local seed funding by recruiting peer reviewers from their own institutional community. Smaller institutions, however, often face difficulty locating qualified local reviewers who are not in conflict with the proposal. As a larger pool of reviewers may be accessed through a cross-institutional collaborative process, nine Clinical and Translational Science Award (CTSA) hubs formed a consortium in 2016 to facilitate reviewer exchanges. Data were collected to evaluate the feasibility and preliminary efficacy of the consortium.

**Methods:**

The CTSA External Reviewer Exchange Consortium (CEREC) has been supported by a custom-built web-based application that facilitates the process and tracks the efficiency and productivity of the exchange.

**Results:**

All nine of the original CEREC members remain actively engaged in the exchange. Between January 2017 and May 2019, CEREC supported the review process for 23 individual calls for proposals. Out of the 412 reviews requested, 368 were received, for a fulfillment ratio of 89.3%. The yield on reviewer invitations has remained consistently high, with approximately one-third of invitations being accepted, and of the reviewers who agreed to provide a review, 88.3% submitted a complete review. Surveys of reviewers and pilot program administrators indicate high satisfaction with the process.

**Conclusions:**

These data indicate that a reviewer exchange consortium is feasible, adds value to participating partners, and is sustainable over time.

## Introduction

The peer review process has long been used to provide “a system of institutionalized vigilance” [1] in the self-regulation of scientific communities. Although there is vigorous debate about how impartial the peer review process is [2], how reliable it is [3], and whether peer review scores are predictive of research productivity [4], the utility of peer review in the evaluation of funding proposals has been repeatedly affirmed [5,6] and the scientific community relies heavily on this process to evaluate competing proposals for funding. One approach to reducing potential sources of bias in peer review is to minimize the degree of affiliation between the applicant and the reviewer and maximize the level of expertise of the reviewer relative to the topic of the proposal [2]. With respect to affiliation, the NIH Center for Scientific Review, in its own peer review process, bars a reviewer from evaluating a proposal if s/he would receive direct financial benefit if the application was funded, is from the same institution as the applicant, or has, within the past 3 years, been a collaborator or has any other professional relationship (e.g., served as a mentor) with any person who has a major role in the proposed research [7].

It is a challenge, however, to apply such rigorous criteria to reviews conducted to inform seed funding decisions within a single research institution or university. One network of NIH-funded research institutes charged with administering a review process to disseminate seed funds is the network of Clinical and Translational Science Awards (CTSAs). As described on the NIH website: “[The CTSA] Program supports a national network of medical research institutions – called hubs – that work together to improve the translational research process to get more treatments to more patients more quickly. The hubs collaborate locally and regionally to catalyze innovation in training, research tools and processes [8].” In fiscal year 2018, there were 58 funded CTSA hubs receiving $459,342,839 from the National Center for Advancing Translational Sciences (NCATS) [9].

Among the mandated components specified in the CTSA, Request for Applications (RFA) [10] is a mechanism for funding translational and clinical pilot studies. Each CTSA hub is afforded considerable flexibility in how pilot studies programs are structured, but certain expectations are established in the RFA, including that the projects should be completed within a 1-year time frame, should not focus exclusively on a single disease, and should be innovative and collaborative. Certain stipulations also are made concerning the process by which pilot studies are to be selected for funding; specifically, a robust review process is required. In recognition of the diversity in possible research topics, “two-level reviews are allowed; a larger group of reviewers with specialized expertise may submit written critiques that are then considered by a smaller multidisciplinary group interacting in real time to make the final decision.”

In practice, there is considerable variability in how individual hubs choose to administer their CTSA pilot studies programs. To capture this diversity, the CTSA hub at the University of California, Irvine (UC Irvine) conducted an online survey of CTSA consortium pilot studies programs in June 2016 (unpublished data). Based on the results of this survey, several common challenges emerged related to pilot studies programs, most notably in implementing a rigorous yet fair review process. Feedback provided on the survey underscored the difficulty in locating qualified reviewers who had professional distance from the applicants. Several institutions attempted “reviewer exchanges” with another CTSA hub. Such arrangements can be very helpful, but also logistically challenging, as there may not be parity across the hubs in terms of the number of applications requiring review and there may be difficulty with harmonizing review calendars. Moreover, the breadth of research topics that may be covered in applications is so vast that any one hub is unlikely to be able to identify willing expert reviewers for every application.

In the fall of 2016, a group of nine CTSA hubs formed a consortium to address the need for locating expert reviewers who were not in conflict with applications submitted for pilot study awards. Participating institutions include: Harvard Catalyst; Medical College of Wisconsin; The Ohio State University; University of Alabama at Birmingham; University of Arkansas for Medical Sciences (UAMS); UC Irvine; University of Southern California; Virginia Commonwealth University; and University of Washington. The CTSA External Reviewer Exchange Consortium (CEREC) has been in continuous operation since that time. As of May 2019, CEREC had facilitated 23 calls for proposals featuring 261 applications. A total of 368 reviews were obtained through the consortium in the period beginning in January 2017 and ending in May 2019. While the innovation described herein was developed to address challenges encountered by pilot study administrators facilitating CTSA-funded pilot programs, the innovation itself, namely the exchange of pertinent, independent, scientific peer reviewers, could be applied to any consortium seeking to access a larger pool of reviewers for the purpose of increasing the rigor of the review process. The exchange model could be applied to pilot programs funded by the NIH P30 mechanism, to the review of applications for institutional NIH K and T awards, or to pilot programs funded through the National Cancer Institute Comprehensive Cancer Centers.

## Methods

### Procedures

CEREC is coordinated by the CTSA hub at the UC Irvine, which schedules monthly consortium conference calls and hosts a web-based interface (referred to as CEREC Central) that allows hubs to post abstracts for proposals requiring reviewers and to update the reviewer matching process from invitation to completion. The process is coordinated yet decentralized, with pilot administrators at the non-requesting institutions extending review requests to investigators at their home CTSAs and then, once a reviewer has agreed to participate, handing over the contact information to the hub that has requested the reviews. Thus, each CEREC hub utilizes its own review process, including how application materials are accessed by the reviewer, how reviewer scores are submitted, and what instructions are provided to reviewers.

During the first year of operations, all efforts devoted to CEREC were incorporated into each hub’s existing personnel job responsibilities. At that time, the management of CEREC was facilitated by an initial version of CEREC Central designed and built by the Director of Biomedical Informatics at the UC Irvine CTSA hub. In the second year, CEREC was awarded 1 year of support from an NCATS administrative supplement. The supplement provided funds for a managing administrator for 9 months to support the coordination of a plan to disseminate the CEREC Model and to create a Manual of Procedures (MOP). The supplement also supported the Biomedical Informatics Director for 0.6 months to further build out CEREC Central, which now features a dashboard, a reviewer database, automatic thank-you emails to reviewers that contain a link to a satisfaction survey, and real-time data on productivity. In addition, each hub administrator was included in the supplement at approximately 16% effort to support the preliminary evaluation of the CEREC model.

### Measures

#### CEREC hub characteristics

Data compiled by the NIH and available on the NIH RePORTER website were accessed to classify hubs according to size, and pilot studies administrators provided data regarding their program characteristics.

#### CEREC member engagement

Once a month, a consortium conference call was held and minutes were transcribed and archived. These minutes provided a measure of engagement, as represented by the attendance records. Additional measures of engagement include the number of proposals posted to CEREC Central by each participating hub and the number of reviews provided to the consortium by each participating hub. These latter two indicators were available from the information automatically archived by CEREC Central.

#### CEREC productivity

Statistics related to the productivity of CEREC activities are automatically tracked by CEREC Central. Productivity was captured as the number of calls for proposals that utilized CEREC, the number of pilot studies proposals that were posted to CEREC Central, the number of reviews requested (some proposals required more than one review), the number of reviews received, and the fulfillment ratio (i.e., percent of reviews requested that were received).

#### Efficiency of the reviewer invitation process

CEREC Central also afforded the ability to track the reviewer invitation process (i.e., the number of reviewers who were invited to contribute a review, the number who declined and/or agreed, and the number who followed through to provide a completed review).

#### Reviewer satisfaction

Toward the end of 2018, an additional feature was added to CEREC Central, which automatically sent each reviewer a link to a survey when the review was logged as completed in CEREC Central. Surveys were managed via the Research Electronic Data Capture (REDCap) [11,12] system. Questions asked included: How satisfied were you with the review process for the external institution? [responses ranged from 1(not at all satisfied) to 4 (extremely satisfied)]; and How likely are you to review for an external institution again? [responses ranged from 1 (not at all likely) to 3 (extremely likely)].

#### Reviewer expertise

The reviewer survey administered during the most recent three calls for proposals also assessed reviewers’ own perceptions of their level of expertise related to the application they reviewed. Two questions were asked: “How qualified did you feel to review this proposal?” and “How confident are you that the review you provided will be helpful for others in evaluating the proposal?” Responses were on a four-point scale from 1 (extremely qualified/confident) to 4 (not at all qualified/confident).

#### Perceived value of CEREC

At the end of 2017 and 2018, a REDCap survey was sent to each of the pilot studies administrators within CEREC. This survey assessed: (1) How satisfied the administrator was with activating CEREC to request proposals and how satisfied the administrator was with the process of providing reviewers to CEREC partners [responses were on a sliding scale from 1 (extremely unsatisfied) to 100 (extremely satisfied)]; (2) How CEREC contributed in a positive way to the institution’s review process (open-ended); and (3) Any benefits that accrued to the institution as a result of participating in CEREC. Possible benefits to the institution were assessed with a checklist, as well as an open-ended item. Items on the checklist included: access to reviewers without a conflict of interest; access to reviewers with needed expertise; access to additional reviewers to supplement internal reviews; access to a wider pool of reviewers than available locally; connected local researchers to the national CTSA; connected local researchers to others doing similar work at other institutions; provided external exposure to local researchers; offered recognition of expertise to local researchers; made us look good to our local constituency; made us look good to our institutional leaders.

## Results

### CEREC hub characteristics

Table 1 illustrates the diversity in pilot study programs across CEREC hubs. Participating hubs are geographically dispersed across the country, vary in size according to NCATS funding qualifications and host pilot programs of variable scope. Owing to differences in the way that hubs administer their programs, the ratio of applications received to projects funded also varies considerably.

**Table 1.** Characteristics of CTSA External Reviewer Exchange Consortium members in calendar year 2018

Hub	CTSA size^1^	No. of RFAs^2^	No. of applications^3^
(1)	Large	2	25
(2)	Small	2	53
(3)	Large	1	36
(4)	Small	2	10
(5)	Small	4	63
(6)	Small	3	50
(7)	Small	2	85
(8)	Small	5	51
(9)	Large	4	110

1 Size is defined by the National Center for Advancing Translational Sciences as follows: small hubs [total anticipated Clinical Translational Science Award (CTSA) amount <$4.5 M DC]; medium hubs (total anticipated CTSA amount $4.5–$6 M DC); and large hubs (total anticipated CTSA amount >$6–$7.5 M DC).

2 RFA, request for applications. This column shows the number of RFAs issued in a usual year wherein the projects are funded either entirely or in part with resources allocated by the CTSA hub.

3 This column shows the total number of applications submitted in response to the RFAs referred to in the previous column.

### CEREC member engagement

The degree to which CEREC members were engaged with the reviewer exchange process is illustrated in Table 2. Participation in monthly telephone conference calls was consistently high. It is interesting to note that the degree to which individual hubs utilized CEREC to identify reviewers for their own RFAs had no apparent relationship to their attendance on the calls. For example, hubs 1 and 9 requested relatively little support from CEREC partners in terms of the number of reviews requested (i.e., 1 and 16, respectively), yet conference call attendance from representatives at these hubs was consistently high. It is also interesting to note that there tends to be a relative balance within a hub between the number of reviews requested and the number of reviews contributed by the hub.

**Table 2.** CTSA External Reviewer Exchange Consortium member engagement (January 2017–March 2019)

Hub	% Conference calls attended	# RFAs supported	# Proposals posted	# Reviews requested	# Reviews contributed
(1)	100	1	1	1	57
(2)	96	2	23	67	47
(3)	88	3	49	49	36
(4)	92	4	34	42	42
(5)	100	4	55	70	67
(6)	92	2	23	32	28
(7)	92	2	33	69	32
(8)	60	3	33	66	39
(9)	76	2	10	16	20

### CEREC productivity

The level of review exchange activity handled by CEREC has remained consistent over time (see Table 3). In the first year, there were 10 RFAs supported by the exchange, and in the second year, 9 RFAs were supported. In the first 4 months of 2019, CEREC supported four RFAs. The projected calendar suggests that the number of RFAs that will be facilitated over the course of the year will be close to 10. Annual fluctuations in the number of RFAs supported are a reflection of the cyclical funding patterns of CTSAs (i.e., hubs must apply for continued funding every 5 years, leading some hubs to experience a lapse in pilot study funding between grant cycles) and of the occasional “special call” that hubs look to CEREC to support. These “special calls” tend to be institutionally funded and related to a specific topic or disease, and typically feature a limited number of proposals. Overall, CEREC has a strong track record of providing reviews, and the preliminary data from 2019 suggest that the process is currently more than 90% effective in supplying external reviews to the CEREC partners.

**Table 3.** CTSA External Reviewer Exchange Consortium productivity

	2017	2018	2019^1^	Total
# of RFAs supported	10	9	4	23
# of proposals posted	97	98	66	261
# of reviews requested	127	157	128	412
# of reviews received	124	125	119	368
Fulfillment ratio^2^	97.6%	79.6%	93%	89.3%

1 Data for the first 4 months of 2019.

2 Fulfillment ratio was calculated as the number of reviews received divided by the number of reviews requested.

### Efficiency of the reviewer invitation process

The data in Table 4 provide insight into the effort that was dedicated to locating expert reviewers for pilot studies applications. On average, about a third of invitations resulted in a completed review, which means that approximately three invitations were issued for every one completed review. These rates have remained fairly consistent over time, with the most recent data from 2019 indicating a very high completion rate (92%).

**Table 4.** Efficiency of the reviewer invitation process

	2017	2018	2019^1^	Total
# Invitations sent	422	453	418	1,293
% Accepted	32.5%	33.6%	30.9%	32.3%
% Declined	35.8%	30.7%	34.4%	33.6%
% Completed^2^	90.5%	82.2%	92.2%	88.3%

1 Data for the first 4 months of 2019.

2 % completed was calculated as number of completed reviews divided by the number who agreed to provide a review.

Note: the percent of invitations accepted and declined do not add to 100 because some potential reviewers failed to respond to the invitation at all.

### Reviewer satisfaction

The data obtained from the reviewer feedback surveys administered after the reviewer submitted a completed review suggested that reviewers were generally satisfied with the process. The survey invitation was emailed to 152 reviewers, of whom 57 completed the survey (response rate = 37.5%). The majority of respondents (68%) indicated that they were extremely satisfied with the review process, and an additional 26% were somewhat satisfied. These ratings were consistent with the proportion of respondents who indicated that they would be very likely (65%) or somewhat likely (33%) to review for an external institution again. When asked to provide comments, several reviewers offered positive affirmations of the process (e.g., “Having this type of consortium for these reviews is extremely helpful. Thanks for supporting this.”).

### Reviewer expertise

Responses to the questions about reviewer expertise suggested that CEREC is effectively meeting the goal of matching qualified reviewers to proposals. Of the 57 respondents to the reviewer survey, the majority (67%) reported that they felt extremely qualified to review their assigned proposal and 33% reported that they felt somewhat qualified to review their assigned proposal. No reviewers indicated that they were “a little” or “not at all” qualified.

### Perceived value of CEREC

At the end of the first year of CEREC operations, pilot studies administrators reported strong satisfaction with the process of activating CEREC to locate reviewers for proposals submitted at their own hub. On average, satisfaction was rated as 89.6 on a scale of 1–100, with responses ranging from a low of 66 to a high of 100. A similar pattern emerged in relation to satisfaction with the process of providing reviewers (mean = 89.78; range = 66–100). When asked on an open-ended question to describe how CEREC had benefitted their review process, pilot studies administrators indicated that the process was achieving its aim of providing access to qualified reviewers (e.g., “Activating CEREC greatly helped in securing reviewers for proposals that we were unable to match using our own reviewer pool. CEREC allows us access to reviewers with expertise that is rare within our local and internal contacts.”).

At the end of the second year of CEREC operations, satisfaction ratings provided by pilot studies administrators were consistently high. Regarding satisfaction with the process of requesting reviews, the average rating was 96, and the range was 90–100. Satisfaction with the process of providing reviews was also high, and the range was smaller than it had been after the first year of operations (mean = 92.5, range = 85–100). As they had in year one, several hubs emphasized the value of obtaining reviews from experts outside their own institution (e.g., “It helped us find reviewers in an efficient manner. And it helped us find reviewers that have absolutely no association with the PIs.”).

Table 5 shows the frequency with which pilot studies administrators endorsed items on a list of possible benefits of CEREC. Pilot studies administrators consistently endorsed the utility of CEREC for providing access to reviewers without a conflict of interest with the proposal being reviewed. Other benefits became more apparent over time, including access to reviewers with needed expertise and access to a wider pool of reviewers than available locally. Administrators also increasingly saw value in connecting local researchers to the national CTSA network and in connecting local researchers to others doing similar work at other institutions.

**Table 5.** Perceived benefits of participation in the CTSA External Reviewer Exchange Consortium (CEREC) (N = 9; % who endorsed each item)

	2017	2018^1^
Access to reviewers without a conflict of interest	100	100
Access to reviewers with needed expertise	88.9	100
Access to additional reviewers to supplement internal reviews	55.6	75
Access to a wider pool of reviewers than available locally	77.8	100
Connected local researchers to the national CTSA network	44.4	66.7
Connected local researcher to others doing similar work at other institutions	55.6	77.8
Provided external exposure to local researchers	66.7	55.6
Offered recognition of expertise to local researchers	66.7	66.7
Made us look good to our local constituency	66.7	44.4
Made us look good to our institutional leaders	77.8	77.8

1 One CEREC member did not request reviews from CEREC in 2018, so the denominator for items related to reviewer requests was 8.

In addition to the benefits related to the purpose for which CEREC was created, ancillary benefits of participating in the consortium also were noted in response to an open-ended question. Comments from administrators included the following:

“The opportunity to learn from other pilot studies programs is invaluable. Often CTSAs on the whole are a bit of a mosaic, without clear instruction or implementation from NCATS. Having other pilot directors to share ideas and solve problems beyond simply reviews is of great benefit.”

“I think it makes it clear that [our hub] is participating in a cross-CTSA collaboration that has tangible results.”

## Discussion

CEREC was formed in response to a perceived need among CTSA pilot studies programs for a systematic method of locating expert reviewers who were not in conflict with submitted applications. Now in its third year of operation, CEREC has built a robust process for meeting this need. All nine of the original CTSA hubs who came together to form the consortium are still actively participating, despite personnel turnovers in some hubs during this time. Subjective reports from pilot studies administrators and consistent engagement over time both suggest that all members are deriving considerable benefit from CEREC participation. In addition, quantitative data from the web-based tool utilized to facilitate the exchanges document the exchange’s consistent productivity over time. Moreover, initial survey responses from reviewers suggest that they find the process easy to use, see the value in obtaining external reviews, and would be willing to review future proposals through CEREC. Reviewer surveys also indicate that CEREC is succeeding in locating reviewers who feel confident in their expertise to review their assigned proposal.

It is important to note that one of the defining characteristics of CEREC is the lack of standardization across programs in terms of how reviews are processed and how external reviews are integrated into the overall review strategy of each hub. For example, hub 9 utilizes CEREC only to identify reviewers for proposals for which qualified and independent reviewers cannot be located within their own hub. That said, this hub has remained actively engaged in the consortium owing to the value of having efficient access to expert reviewers for the inevitable group of proposals for which internal reviewers are not available. In contrast, hub 2 utilizes only external reviewers for their pilot studies reviews. Their reliance on CEREC, therefore, is relatively high and is balanced by their considerable effort to procure reviewers for their CEREC partners. Helping to ensure a balance over time, CEREC Central offers a feature for tracking each hub’s standing in terms of “credits” and “debits” to the consortium, and this public accountability promotes a relative parity of effort over time.

In 2018, CEREC was awarded an administrative supplement by NCATS to support a further build-out of CEREC Central, a preliminary evaluation of CEREC efficacy, and dissemination of the CEREC model. With this support, CEREC Central was revamped and a face-to-face meeting of CEREC partners was convened to provide input to the revision and to generate a MOP. The result has been a considerably enhanced product that streamlines the process in a number of ways and may explain the apparently improved efficiency in 2019.

In addition to fulfilling its original promise to support and enhance the reviewer exchange process, CEREC also has led to a general exchange of best practices between and among the participating hubs. Innovations developed at one hub have been showcased to the consortium and some CEREC partners have adopted these innovations. For example, the Medical College of Wisconsin leveraged REDCap to manage their reviewer database and to integrate this database into their application and review process. To date, two CEREC partners have adopted this approach, and at least one more has plans to do so. In addition, The Ohio State University CTSA pilot studies program adopted an innovation developed by the UAMS for integrating community members into the application review process through a Community Scientist Academy. The latter transfer of knowledge was further developed into an administrative supplement awarded to a subset of the CEREC hubs to disseminate and evaluate the UAMS approach for integrating community-based reviewers into the review process.

In accordance with the NCATS mandate to “Develop, Demonstrate, Disseminate,” the members of CEREC are now recruiting a new group of CTSA hubs with the intention of mentoring them through the process of establishing a CEREC II. To that end, we have compiled a MOP, and the UC Irvine hub will dedicate personnel efforts to mentoring CEREC II. Once the utility of the MOP has been demonstrated through the successful dissemination of the CEREC model to a second consortium, the MOP will be made publicly available. Internal discussions among CEREC members generated a consensus opinion that dissemination would be best accomplished by replicating the model, rather than by adding new members, for several reasons. Firstly, the success of the CEREC process draws heavily on a personal sense of obligation among members to contribute to the partnership; too large a consortium would dilute that sense of personal obligation. Secondly, the current size of the consortium results in a manageable ebb and flow of demand on the consortium, with periods of little to no activity, during which members can focus internally on their own programmatic activities, and periods of strain, during which members are being asked to simultaneously attend to the needs of multiple CEREC partners. Adding more hubs to CEREC would risk straining the partnership to the point of breakage.

Central to the effort to disseminate the CEREC model will be the identification of a hub that is willing to take on a coordinating role. Our experience has demonstrated that there needs to be a single hub that assumes the responsibility for scheduling consortium conference calls, managing the harmonized calendar of requests for proposals, and monitoring the progress of each CEREC activation. This administrative demand must, of course, be endorsed by the hub PI, who must be willing to allocate administrative effort to the demands of the consortium coordination. Members of CEREC have had little difficulty persuading their PIs of CEREC’s added value, given that it is in line with the review criterion spelled out in the NIH Request for Proposals and reflects one of the objectives of the strategic plan of the NCATS “share resources and expertise across the federal government through collaborative research, particularly with other NIH Institutes and Centers [13].” It should be noted that this NCATS goal explicitly broadens the potential for replication beyond the CTSA network and future dissemination might engage cross-institutional collaborations among NIH-funded training programs, cancer institutes, or other multi-institutional seed funding programs.

CEREC has effectively addressed the need to recruit qualified reviewers who are not in conflict with submitted proposals in order to yield a robust assessment of scientific merit. The longevity of the consortium, however, can be attributed to the perceptions of the members that participation confers added value to individual hubs above and beyond the reviewer exchange. Ancillary benefits include access to a ready network of colleagues available to provide quick feedback to members who encounter a challenge in program administration, mentoring of new pilot studies administrators following personnel transitions, and the reward that comes from having one’s own innovations adopted by partner institutions (e.g., The Ohio State University CTSAs practice of providing reviewers with a formal thank-you letter that may be useful for promotion reviews). As a result, CEREC appears to be a very sustainable model for reviewer exchange, and one that could potentially be expanded to additional functions, such as the review of mentored scientist (KL2) applications.

In addition to the clear benefits of CEREC, there also have been a number of challenges. As mentioned earlier, each participating hub has the freedom to administer their review process in a manner that is decided locally, thus introducing considerable diversity. A minority of the participating hubs, for example, require that external reviewers participate remotely in the face-to-face discussion which comprises the final stage of the review process. Coordinating this participation, particularly across several time zones, can be difficult, and generally results in greater difficulty locating reviewers. Data from CEREC Central indicate that the fulfillment ratio for one site that requires study section participation was 51%, compared to the 89.3% overall. In these cases, therefore, CEREC administrators are encouraged to embark on the reviewer identification process without delay to allow for the possibility of more invitations being declined. Another challenge is the unavoidable overlap of multiple CEREC activations simultaneously, resulting in some participating administrators being pressed for time in locating reviewers for a CEREC partner. One strategy that we have developed to compensate for these times is to have the requesting hub administrator carry out online searches for potential reviewers at a CEREC partner’s institution and then forward those contacts to the providing hub administrator who issues the invitations locally. Despite the best efforts of all, CEREC does occasionally fail to procure a review for a given proposal. For these cases, local pilot program administrators typically maintain a few go-to emergency reviewers at their local hub to whom they can turn to provide a last-minute review if need be. Each of the challenges that have been encountered has led to a tightening in local and consortium practices so that the result is the continuous improvement of the pilot funding application review process.

As noted in the results section, the vast majority of reviewers who responded to the post-review survey were very satisfied with the experience, but there were some suggestions for improvement. Among the most common suggestions were requests for a link to the Request for Funding associated with the application, simplification of the scoring instructions, and the opportunity to provide more qualitative feedback (as opposed to just number scores). Each of the participating CEREC partners utilizes a unique system for obtaining reviews, so these comments reflect individual reviewers’ perceptions of the process at an individual hub. Methods varied considerably. One hub emails the application and review materials directly to the reviewer and receives responses via email. A few sites use REDCap to send and receive the information, and others employ home-grown web-based systems. Feedback from the reviewer surveys is periodically shared with each site to inform quality improvement efforts.

One limitation of the data presented herein is that there are no current benchmarks for evaluating the effectiveness of CEREC in relation to other review-finding processes. For example, the fulfillment ratio of 87.9% indicating that almost 90% of reviewers turn in their review appears like a very favorable number, but we have no standards against which to compare. None of our collaborating partners systematically tracked this information prior to joining CEREC, so comparative numbers were not readily available. Another limitation is that the hubs participating in this exchange were self-selected for high interest in establishing this type of collaboration. Whether such an exchange could be successful if it did not arise organically out of a shared perception of need is as of yet an untested question. However, the fact that during the lifetime of CEREC, there has been administrative turnover at four of our participating institutions suggests at least that the potential goes beyond the personal commitment of individual program administrators. It is important to note that the data reported here are descriptive in nature and the generalizability of CEREC as a model is yet unknown.

A possible future contribution of CEREC is to examine alternative approaches to selecting proposals for funding. Considerable hub resources are devoted to the review process across the entire CTSA national network; a fact that reflects the generally held confidence in peer review as a method of evaluating research funding proposals [5,6]. A small but growing literature has begun to investigate the role and dynamics of peer review in the funding decision process, and there has been a call for more systematic research to identify optimal processes for selecting science for funding that is both innovative and likely to be successfully executed [14,15]. One such study concluded that there was no substantial improvement in how accurately mean reviewer scores predicted funding decisions when the number of reviewers increased above four [14]. Others have evaluated the impact on the review process of simplifying the application materials; a modification which has met with reviewer approval, but has uncertain impact on the outcome of the review process [6,16]. Of particular interest is the role of the face-to-face reviewer discussion in identifying meritorious projects that have advanced beyond the numerical rating stage. There is considerable debate about the added value of this second phase to the review process [4], with some academics suggesting that conferring awards by lottery (among the top-scoring proposals) might make the process more efficient, require fewer resources, and result in the funding of projects that have no less likely a probability of generating high-impact results [15–18]. One extension of the current research would be to leverage CEREC to conduct systematic investigations to inform the review process so as to maximize the likelihood that funded projects will be completed successfully and will advance the scientific enterprise. Another extension would be to consider the CEREC database as a repository of funded and unfunded pilot abstracts that may be evaluated further for trends, themes and activities in preliminary clinical and translational pilot studies within a sampling of CTSA institutions.
